# A case of posterior reversible encephalopathy syndrome during endoscopic retrograde cholangiopancreatography after anesthesia 

**Published:** 2022

**Authors:** Amir Sadeghi, Isa Bakhshandeh Moghadam, Azita Hekmatdoost, Niloufar Salehi, Mohammad Reza Zali

**Affiliations:** 1 *Gastroenterology and Liver Disease Research Center, Research Institute for Gastroenterology and Liver Diseases, Shahid Beheshti University of Medical Sciences, Tehran, Iran*; 2 *Department of Neurology, Taleghani Hospital, Shahid Beheshti University of Medical Sciences, Tehran, Iran*; 3 *Department of Clinical Nutrition and Dietetics, Faculty of Nutrition and Food Technology, National Nutrition and Food Technology Research Institute, Shahid Beheshti University of Medical Sciences, Tehran, Iran*

**Keywords:** PRES, Opioid, Fentanyl, MRI, Case Report

## Abstract

Posterior reversible encephalopathy syndrome (PRES) is a neurological disorder that occurs following cerebral vasogenic edema. It has diverse clinical presentations from headache and vomiting to seizure and mental status alteration. Herein, we report a 54-year-old woman with no prior disease who developed PRES in the parieto-occipital lobes and brain stem after a second attempt endoscopic retrograde cholangiopancreatography (ERCP). To our knowledge, no case of PRES during ERCP has been reported to date. This case reminds us of unusual complications that are likely to occur after ERCP. It is believed that blood pressure fluctuations and anesthetic medications, fentanyl in particular, were the main precipitating factors causing the syndrome in the current case. Even if there is no specific treatment for this condition, a diagnosis is critical to start supportive treatment.

## Introduction

 Endoscopic retrograde cholangiopancreatography (ERCP) is a minimally invasive procedure routinely used for the diagnosis and treatment of pancreatobiliary pathologies. Due to the increased usage of this technique, complications occur in 7-10% of patients undergoing ERCP (1). The most common complications are pancreatitis (1-5%), cholangitis (1-5%), perforation (1-2%), and hemorrhage (1%) (2). Some rare cases of acute confusional states after ERCP have also been reported from which most have had brain infarction following cerebral embolism (3). 

Posterior reversible encephalopathy syndrome (PRES) is a condition occurring due to cerebral vasogenic edema. It has specific neuro-radiological characteristics which are usually reversible (4). Herein we present a case of acute confusional state during a second attempt ERCP that was diagnosed with PRES. To our knowledge, no case of PRES during ERCP has been reported to date. The patient was handled at an academic hospital. 

## Case Report

**Figure 1:**
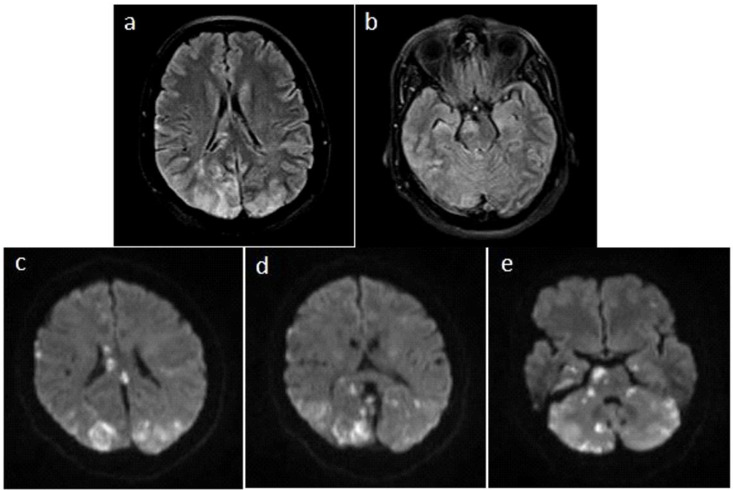
Axial ﬂuid-attenuated inversion recovery (FLAIR) (a, b) and diffusion-weighted (c, d, e) magnetic resonance images 2 days after presentation indicates increased signal intensity in parietooccipital lobes and pons

A 54-year-old woman with no significant past medical history transferred to our specialized center following jaundice, nausea, and epigastric pain in addition to raised cholestase parameters (total and direct bilirubin, SGOT, SGPT, and alkaline phosphatase). Dilated intrahepatic bile duct and a filling defect (18*15 mm) in the middle part of CBD were discovered in magnetic resonance cholangiopancreatography. The patient underwent ERCP. Sphincterotomy and stenting were conducted. Remnant stone was reported, so she was advised to return for a second ERCP after 2 months, but she came later, i.e. after 7 months. Ultrasonography detected a hyperecho intraluminal defect that had not blocked the duct to the fullest, and the stent was exactly distal to the defect. Laboratory tests were normal. In repeated ERCP, duodenoscopy and removal of previous stent was done. CBD was 20 mm and contained one 15-mm-long stone in the proximal part. Dilation with balloon TTS was conducted, and the large stone was removed by use of the balloon. Bleeding in the amount of 100-150 cc was seen and stopped after water insufflation. One plastic stent was inserted, and consequently, complete drainage was observed. At the commencement of the procedure, the patient’s blood pressure (BP) was 160/100, but it was reduced to 130/80 using anesthetic techniques and agents. After blood loss, BP dropped to 100/60 but later came under control and rose to 130/80 again. Meanwhile, the patient had a normal breathing pattern yet experienced a drop in O_2_ saturation from 100% to 88%. Other vital signs were in normal range. When the procedure was completed and the effect of the anesthesiologic medications (propofol 100 mg, midazolam 1 mg, and fentanyl 1 mg) vanished, the patient did not come back to consciousness. She had a Glasgow Coma Scale (GCS) score of 6/15 and merely had withdrawal responses to pain to the right side of her body, but she showed no motor response with painful stimulation to the left side. For better care and further evaluation, the patient was transferred to the intensive care unit. At that point, oxygen saturation was 99% with oxygen mask with reservoir and high O_2_ flow (8-10 L/min), and BP was 130/80. In laboratory tests, hypomagnesaemia (Mg=1.2 (1.4-2.5)) was identified, and arterial blood gas analysis showed a mild respiratory acidosis. Other laboratory findings were normal. The patient having stabilized, gastroscopy was performed to rule out post-ERCP bleeding. No active bleeding was seen. Then the patient received a spiral chest and brain computed tomography (CT) scan that manifested a bilateral plural effusion, yet no pathology in the brain was detected. Echocardiography was also performed and showed a left ventricular ejection fraction of 55% with no anatomical abnormality. Standard and diffusion-weighted brain magnetic resonance imaging (MRI) was taken 2 days later and revealed increased signal intensity representing vasogenic edema in the supratentorial (both parieto-occipital lobes) and infratentorial (mostly the pons) regions (Figure 1). The location of the edema and its bilateral nature indicated PRES. During hospitalization, the patient continually received supportive treatment. In the first 11 days significant enhancement in neurological symptoms was identified that matched with a PRES prognosis. She had eye opening to verbal commands and pain withdrawal. Then the patient was transferred to the gastrointestinal ward. After 20 days she was discharged from the hospital with a GCS of 13/15.

## Discussion

PRES was first defined by Hinchey *et al.* in 1996. The common precipitating factors initially recognized for this syndrome include acute hypertension, renal disorder, fluid retention, and immunosuppressive therapy (5). Since then, myriad reports describing PRES have been published and a variety of causes have been associated with it (4, 6). Two main hypotheses have been proposed as the pathophysiology of PRES. The first theory suggests hypertensive crisis as the main cause of most cases of PRES. Cerebral perfusion pressure in normal individuals is between 50-150 mmHg. This continuous blood flow is preserved by the cerebrovascular autoregulatory system. A severe hypertension or rapid fluctuations in BP might change this autoregulatory threshold and cause extravasation of plasma and edema. Moreover, the vasoconstriction that occurs following hypertension leads to a reduction in brain perfusion, ischemia, and consequently, vasogenic edema (7, 8). The second theory posits that PRES could also be triggered by endothelial dysfunction. Endogenous and exogenous toxins (e.g., cytotoxic medications) can disrupt the endothelial integrity directly or by stimulating the release of vasoactive and immunogenic agents and cause edema formation (4). In the present case, the cause of this syndrome has not been completely elucidated. It is assumed that the severe BP fluctuation during the procedure played a crucial role in PRES development. On the other hand, pharmacotoxic side effects of the anesthetic medications should also be considered. One case of PRES after anesthesia with propofol, ketamine, and fentanyl in a hypertensive patient has been reported, but the association of the syndrome and general anesthesia was not fully demonstrated (9). Moreover, fentanyl, which was used for our patient as a pain relief agent, is a synthetic opioid. Few reports have been published on the incidence of PRES because of opioids (10-12). Eran *et al.* believed there was a connection between PRES and intrathecal morphine administration (10). Additionally, PRES has been confirmed in three patients with methadone toxicity (11).

The clinical symptoms of PRES vary markedly depending on the extension and location of the brain lesions and might develop within the range of hours to days. The most common manifestations are headache, nausea and vomiting, visual disturbance, focal or generalized seizure, and altered level of consciousness ranging from drowsiness to coma (4, 13). Status epilepticus has also been reported as a rare yet severe and life- threatening complication of this syndrome (14). Due to the non-specific presentations, the definitive diagnosis is made after evaluating neuroimaging findings and excluding other diagnoses. Lesions might be detected in non-contrast CT scan in some patients, but MRI, especially T2 weighted fluid-attenuated inversion recovery (FLAIR), is the choice imaging when searching for PRES radiologic findings (15). Diffusion-weighted imaging (DWI) is mandatory and allows for a clearer differentiation between vasogenic edema and other cerebral diseases such as infarction (15, 16). The most common site of brain lesions in PRES is occipital lobes with 31% extension to parietal lobes and rarely temporal lobes change. Frontal lobes edema was also found to have a high incidence. Less common cases had involvement in cerebellum, brain stem, and basal ganglia (17). Electroencephalography (EEG) is useful in assessing encephalopathy. It is also required for monitoring non-convulsive seizures (18). Zou al. established a scoring scale helpful for the early detection of PRES which has three parts: risk factors, clinical presentations, and EEG features (19). Laboratory test results in PRES are mostly non-specific. Hypomagnesaemia have been found in some patients (20). The current case had mild hypomagnesaemia in the first 48 hours as well. 

Regarding the acute neurological deterioration in our patient, possible differential diagnoses include intracranial infarction or hemorrhage, infections and sepsis, deep sedation, imbalanced blood sugar, electrolyte disorders, hypoxia, and inadequate pain management (21, 22). The clinical presentations plus laboratory and MRI findings along with the reversibility of clinical manifestations in the current case all together confirmed the diagnosis of PRES.

As the neurological presentations are mostly reversible, the prognosis of this syndrome is usually favorable, unless it has been associated with grueling complications. The treatment of PRES is based on eliminating or controlling the underlying causes (4), but if no clear cause is found, it is arduous to manage the disease, and it could even be fatal (23). Awareness of this syndrome and knowledge of the risk factors and diagnostic findings help physicians minimize the occurrence or prevent further morbidity and mortality by early detection.

## Conflict of interests

The authors declare that they have no conflict of interest.
